# Resynchronization and defibrillator therapy with a single left bundle branch area defibrillation lead in isolated persistent left superior vena cava

**DOI:** 10.1093/ehjcr/ytag585

**Published:** 2026-08-03

**Authors:** Alexander Steger, Martina Steinmaurer, Andras Fendt, Eimo Martens

**Affiliations:** Department of Internal Medicine I, TUM University Hospital, Technical University of Munich, Ismaninger Straße 22, 81675 Munich, Germany; German Centre for Cardiovascular Research (DZHK), Partner Site Munich Heart Alliance, Munich, Germany; Department of Internal Medicine I, TUM University Hospital, Technical University of Munich, Ismaninger Straße 22, 81675 Munich, Germany; Department of Heart Rhythm Disorders, Barmherzige Brüder Hospital, Prüfeninger Straße 86, 93049 Regensburg, Germany; Department of Internal Medicine I, TUM University Hospital, Technical University of Munich, Ismaninger Straße 22, 81675 Munich, Germany; European Reference Network for Rare and Low Prevalence Complex Diseases of the Heart (ERN GUARDHeart), Munich Consortium for Rare Heart Disease, Munich, Germany

**Keywords:** Isolated persistent left superior vena cava, Left bundle branch area pacing, Cardiac resynchronization, Implantable cardioverter defibrillator, Sudden cardiac death prevention

A 65-year-old man with post-myocarditis cardiomyopathy (left ventricular ejection fraction 21%), permanent atrial fibrillation, and complete left bundle branch block (QRS-duration 160 ms) was referred for cardiac resynchronization therapy and ICD-implantation. Magnetic resonance imaging revealed an isolated persistent left superior vena cava (iPLSVC), with absence of the right superior vena cava and drainage into a dilated coronary sinus **(*Panel A*; asterisk; LPA, left pulmonary artery; DA, descending aorta)**. The anomaly was confirmed by venography **(*Panel B*; asterisk)**. A suitable coronary sinus branch for left ventricular lead placement was not identified. Therefore, a conduction system pacing strategy was chosen. Through a left axillary venous access, a dedicated delivery catheter (Abbott CPS Direct Universal 3D) was advanced via the iPLSCV, coronary sinus, and right atrium into the right ventricle. A mid-septal position targeting the left bundle branch area was confirmed by contrast injection **(*Panel C*)**. An Abbott Durata single coil defibrillation-lead was then deeply screwed into the interventricular septum (off-label use). The procedure was completed without complications using a single-chamber cardioverter-defibrillator **(*Panel D*)**. In the final position the paced QRS-duration decreased to 125 ms **(*Panel E*)**. Defibrillation-testing demonstrated appropriate detection of induced ventricular fibrillation and successful termination by a 30-Joule shock, restoring sinus rhythm **(*Panel F*)**. Subsequent device interrogation confirmed appropriate lead and device function.

PLSVC occurs in approximately 0.2%–0.5% of the general population. Absence of the right superior vena cava represents a rare subtype that may complicate transvenous device implantation.^1–5^ This case illustrates the feasibility of implanting a conventional defibrillation-lead in the left bundle branch area in the setting of iPLSVC, achieving electrical resynchronization and reliable defibrillator function.

**Figure:**
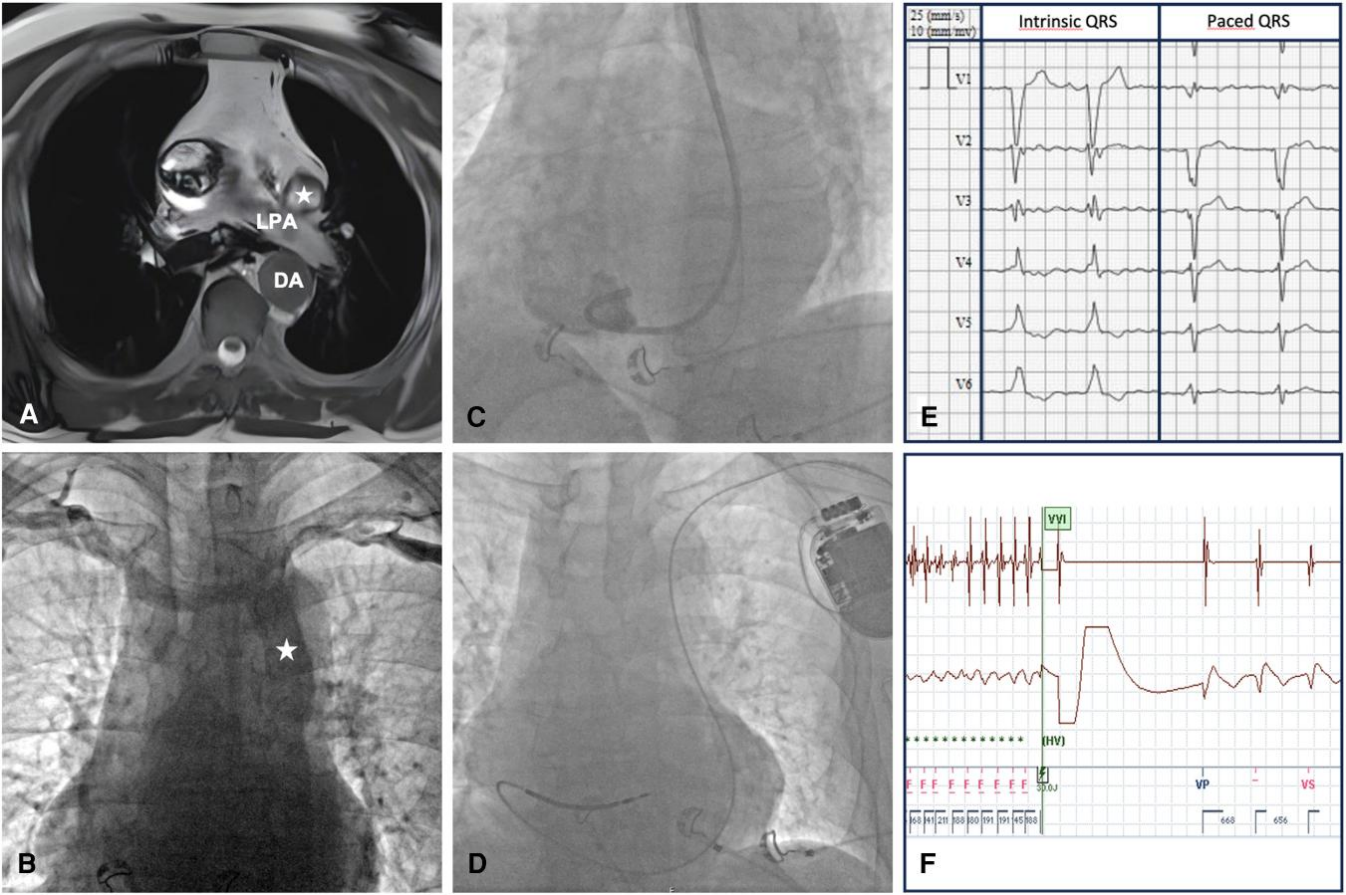


## Data Availability

The de-identified clinical data underlying this article will be shared on reasonable request to the corresponding author.
